# Short-Term Effectiveness of Web-Based Guided Self-Help for Phobic Outpatients: Randomized Controlled Trial

**DOI:** 10.2196/jmir.3429

**Published:** 2014-09-29

**Authors:** Robin N Kok, Annemieke van Straten, Aartjan T F Beekman, Pim Cuijpers

**Affiliations:** ^1^Department of Clinical Psychology and the EMGO institute for Health and Care ResearchVU University AmsterdamAmsterdamNetherlands; ^2^National Institute for Mental Health ResearchThe Australian National UniversityCanberraAustralia; ^3^Department of Psychiatry and the EMGO institute for Health and Care ResearchVU University Medical CentreAmsterdamNetherlands; ^4^Innovation IncubatorDivision Health-Training.OnlineLeuphana UniversityLüneburgGermany

**Keywords:** phobias, phobic disorders, anxiety disorders, Web-based intervention, Internet therapy, randomized controlled trial, outpatients

## Abstract

**Background:**

Internet-based guided self-help has been successfully used in the general population, but it is unknown whether this method can be effectively used in outpatient clinics for patients waiting for face-to-face psychotherapy for phobias.

**Objective:**

The aim was to assess the clinical effectiveness of Phobias Under Control, an Internet-based intervention based on exposure therapy with weekly guidance.

**Methods:**

We conducted a randomized controlled trial, recruiting 212 outpatients scheduled to receive face-to-face psychotherapy for any type of phobia at an outpatient clinic. Participants suffering from at least 1 DSM-IV or ICD-10 classified phobia (social phobia, agoraphobia with or without panic disorder, and/or specific phobia as ascertained by a telephone interview at baseline) were randomly allocated to either a 5-week Internet-based guided self-help program based on exposure therapy with weekly student support followed by face-to-face psychotherapy (n=105) or a wait-list control group followed by face-to-face psychotherapy (n=107). Primary outcome was the Fear Questionnaire (FQ). Secondary outcomes were the Beck Anxiety Inventory (BAI) and Center of Epidemiological Studies-Depression scale (CES-D). Assessments took place by telephone at baseline (T0) and on the Internet at posttest (T1, self-assessment at 5 weeks after baseline). Missing data at T1 were imputed.

**Results:**

At posttest, analysis of covariance on the intention-to-treat sample showed significant but small effect sizes between intervention and control groups on the FQ (d=0.35, *P*=.02), CES-D (d=0.34, *P*=.03), and a nonsignificant effect size on the BAI (d=0.28. *P*=.05). Although initial acceptance was good, high nonresponse was observed, with 86 of 212 participants (40.5%) lost to follow-up at T1 and only 14 of 105 (13.3%) intervention participants finishing all 5 weeks.

**Conclusions:**

Phobias Under Control is modestly effective in lowering phobic and depressive symptoms in a relatively short period and may be clinically beneficial when implemented in routine outpatient practice.

**Trial Registration:**

Netherlands Trial Register NTR2233; http://www.trialregister.nl/trialreg/admin/rctview.asp?TC=2233 (Archived by WebCite at http://www.webcitation.org/6O2ioOQSs).

## Introduction

Phobias are among the most common mental disorders and the most common type of anxiety disorders [1]. Specific phobias are the most common form of anxiety disorders for both genders, with a total 12-month prevalence of 7.1%, followed by social phobia (4.8%) and agoraphobia without panic disorder (1.2%). All phobias have a negative impact on quality of life and psychosocial functioning [2], and the societal burden of phobias is considerable [3,4]. Despite detrimental effects on quality of life, research has shown a substantial delay of more than 10 years between onset of symptoms and first therapy attendance [5]. In a recent study, social phobia was found not only to have the earliest onset age, but also an even longer delay—on average 28 years—in seeking treatment [6]. Notwithstanding the impact of a phobia on a patient’s quality of life [2,7], phobias are often not the primary reason for seeking treatment from an outpatient clinic [8,9] and it has been argued that commonly occurring comorbid disorders, such as depression, mask underlying social phobia leading to underdiagnosis in primary care [10]. This suggests widespread undertreatment [11] for these disorders, even though there is robust evidence of efficacious psychological treatments for agoraphobia [12], social phobia [13], and specific phobias [14], most notably exposure therapy and cognitive behavioral therapy (CBT).

Internet-based interventions are increasingly popular adaptations of evidence-based psychotherapies as a replacement of, or adjunct to, traditional face-to-face therapies. Starting with computer-based, offline interventions (eg, [15,16]), existing therapies such as CBT, exposure therapy, systematic desensitization, and relaxation were found to be efficacious [17] and were rewritten to suit delivery on the Internet [18,19]. In past years, Internet interventions have been found efficacious for a number of anxiety disorders [17,18,20,21] and phobias, including agoraphobia [22], specific phobias [14,23,24], and social anxiety disorder [25-27]. Thus, Internet-delivered psychological treatments for anxiety and phobias are feasible, acceptable, and effective.

Typically, outpatients exhibit higher levels of anxiety and a greater number of comorbid and more complex diagnoses, as well as greater psychosocial impairment when compared with general and primary care populations [5]. Previous research has primarily focused on self-referred participants from primary care settings or from the general population [28], and although some evidence exists on the effectiveness of routine psychological interventions in outpatients [29], only a limited number of trials have specifically evaluated Internet-based treatments in outpatient clinics and secondary care for common mental disorders [30-33]. To the best of our knowledge, there appear to be no large-scale high-quality trials evaluating the efficacy of Internet-based exposure therapy in phobic outpatients.

Because waiting lists are commonplace in outpatient clinics, time spent waiting for face-to-face treatment could be spent effectively by offering a (guided) self-help intervention to patients. Delegating the routine, basic elements of exposure treatment to a guided Internet-based situation could shorten face-to-face therapy and limit therapist involvement, making the treatment more cost-effective [19,34]. Previous research has indicated that Internet-based therapy for social phobia might be cost-effective relative to face-to-face therapy [35,36]. Furthermore, because pretreatment dropout is common in outpatient clinics [37], a second postulated benefit may be that continually engaging the patients in their treatments throughout the wait-list period will result in lower pretreatment attrition or “no shows.”

The objective of the current trial was to assess the short-term clinical effectiveness of offering Internet-based guided self-help to outpatients compared to a wait-list control. To our knowledge, this is the first large-scale randomized controlled trial of Internet-based treatment for phobias in outpatients. As such, it will also provide valuable information on the acceptability and feasibility of such an intervention in outpatient clinics. This paper describes the principal short-term outcomes of this multifaceted trial.

## Methods

### Trial Design

A full trial protocol is available elsewhere [38]. This trial was approved by the Medical Ethics Committee of the VU Medical Centre, Amsterdam (registration number 2010/77) and registered with the Dutch Trial Registry (NTR2233). A total of 481 participants who recently applied for psychological treatment at an outpatient clinic consented to be contacted by our research group and were referred to the researchers from August 2010 to December 2013. After briefing the participants about the aims of the study, screening, and obtaining informed consent in writing, eligible participants (n=212) were administered a telephone baseline questionnaire and participants were randomized to either the intervention group (n=105) or treatment as usual (n=107). Patients who were ineligible (n=111), declined participation, or could not be contacted (n=153) remained on the waiting list for face-to-face treatment. The research did not interfere with the outpatient clinics’ wait-list duration or start of treatment and participants could start face-to-face psychotherapy after the intervention or control group period.

### Participants

#### Recruitment Procedure

A total of 8 specialized anxiety disorder outpatient clinics in medium-to-large cities in the west of the Netherlands participated. Clinics were selected for a high monthly volume of patients for practical reasons. Participants were referred to the outpatient clinics by their general practitioners (GPs), briefly screened, and placed on a waiting list. Recruitment commenced in August 2010 and was stopped in December 2013 to allow for sufficient follow-up time. Waiting lists for outpatient psychotherapy are common in the Netherlands, and time spent on a waiting list is usually at least 6 weeks from first referral to first treatment session. At the start of the wait-list period, participants presenting with a phobia as a primary or secondary disorder were referred to the researchers and screened by telephone using the Composite International Diagnostic Interview (CIDI) [39] for presence of any phobia by master’s level students. Consequently, exclusion criteria were checked and baseline measures were administered. During this wait-list period, a nontherapeutic meeting with a health care professional from the outpatient clinic took place to ascertain treatment needs and to determine optimal face-to-face treatment for all participants. Additional details on recruitment are available elsewhere [38].

#### Eligibility Criteria for Participants

All computer-literate patients with a possible phobia (social phobia, agoraphobia with or without panic disorder, specific phobia) were referred to the researchers by the outpatient clinic even if a phobia was not the primary reason for seeking treatment at an outpatient clinic. Participants had to (1) be 18 years or older, (2) be currently enrolled to receive face-to-face psychotherapy at 1 of the participating outpatient clinics, and (3) have a *Diagnostic and Statistical Manual of Mental Disorders* (Fourth Edition, Text Revision; *DSM-IV-TR*) or *International Classification of Diseases, Tenth Revision* (*ICD-10*) diagnosis of any phobia as established by the CIDI. Psychotropic medication use was allowed if stable for at least the duration of the intervention or control group period. Patients presenting with psychotic disorders or at elevated risk for suicide were excluded from the trial, but remained on the waiting list for face-to-face psychotherapy at their outpatient clinics.

### Interventions

#### Internet-Based Guided Self-Help

The Internet-based intervention is an adaptation of an existing self-help book on phobias [40]. The intervention is offered at no cost to the participant, takes 5 weeks to complete, and is based on psychoeducation and exposure therapy. The broad and nonspecific focus of the intervention is on identifying and correcting avoidance behavior by using exposure, a common and evidence-based therapeutic component of most phobia therapies [41]. This broad focus facilitates using the intervention for the entire range of phobias. The intervention was presented to the prospective participants as a free-of-charge voluntary course to start reducing their phobic symptoms during the wait-list time. They were told that the intervention was based on evidence-based principles and that the elements they would encounter during the intervention would essentially be the same as in their upcoming face-to-face psychotherapy, allowing for a head start in their treatment. Participants were informed that face-to-face treatment would commence at the scheduled time, regardless of whether they enrolled in the study, and that their decision to participate or not would neither postpone nor advance their face-to-face treatment.

**Figure 1:**
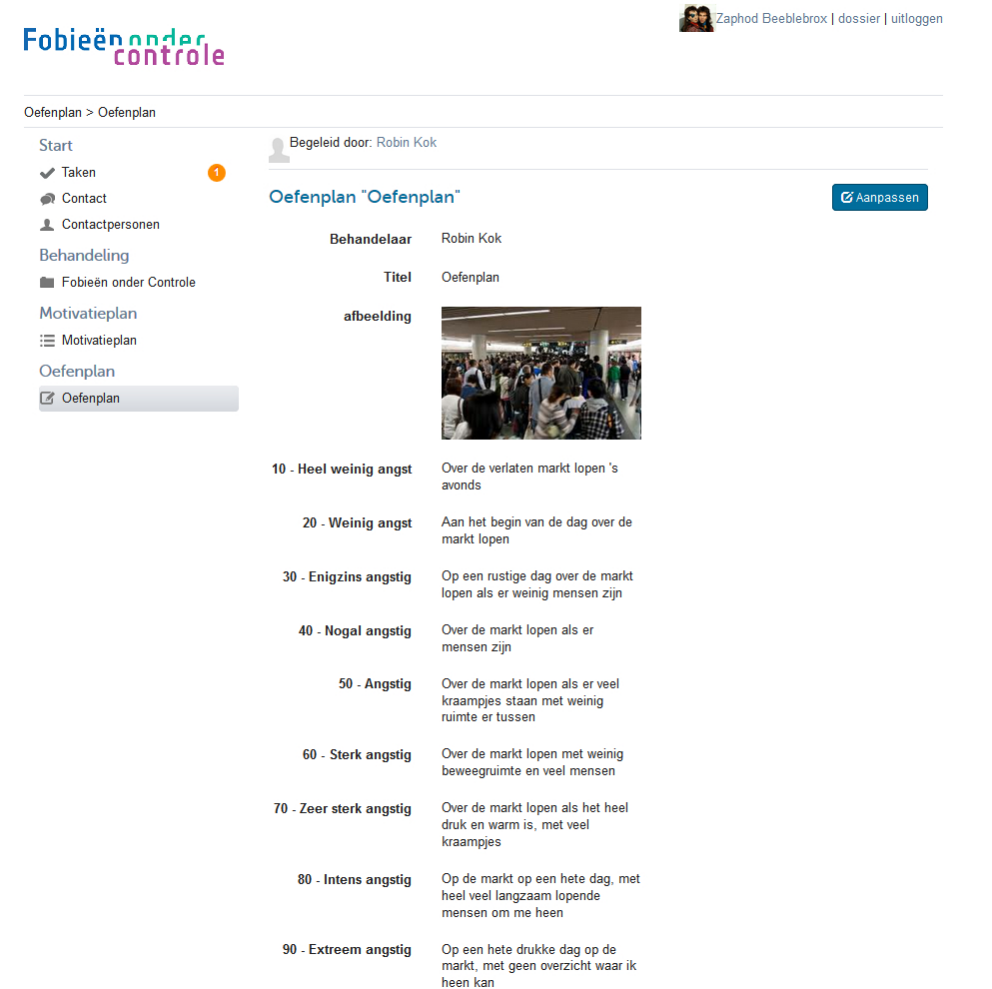
Screenshot of Phobias Under Control: fear hierarchy.

**Figure 2:**
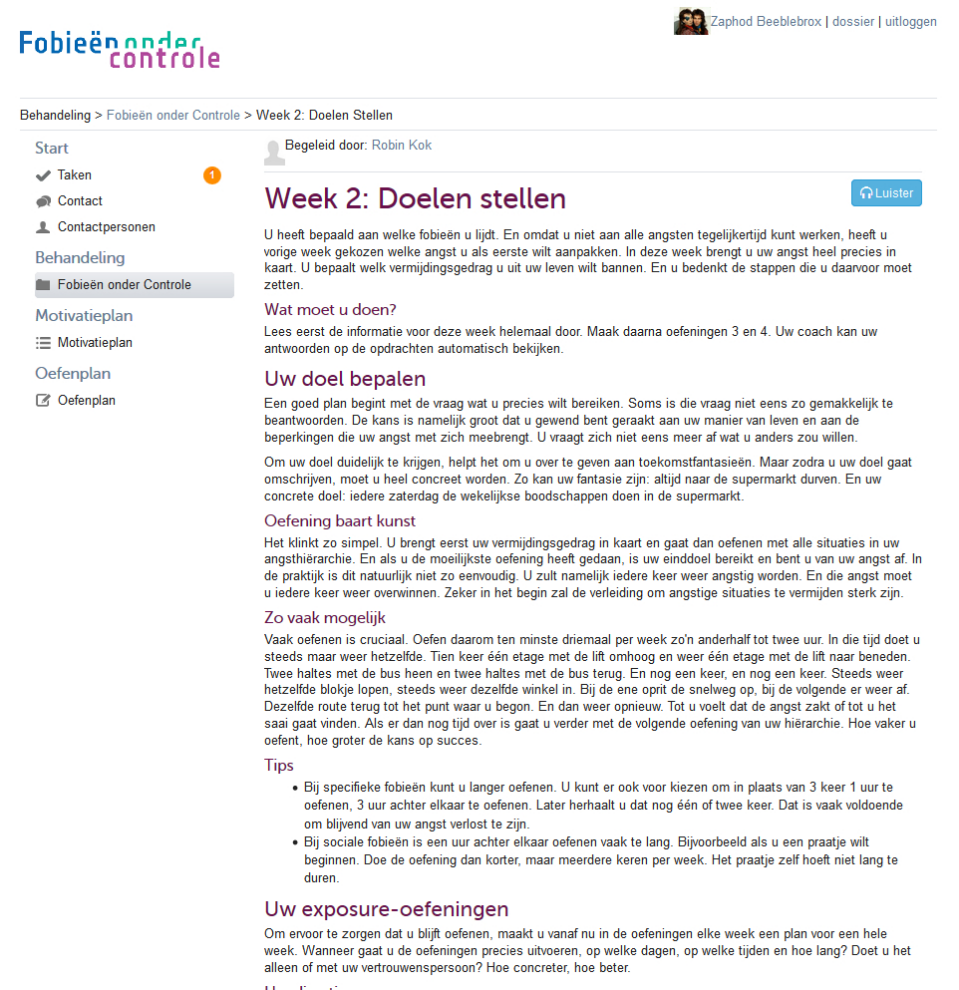
Screenshot of Phobias Under Control: intervention content.

During the intervention, participants build a hierarchy (see Figure 1) of fear-inducing situations or stimuli and expose themselves to these situations or stimuli gradually. The participant completes exposure exercises as homework assignments and reports on his or her accomplishments to the coach each week. In the first weeks, the participant makes an inventory of his or her avoidance and safety behaviors and defines a focal point for exposure situations and a desired behavioral goal. The participant then plans a number of gradual exposure exercises to be executed for the upcoming week, with exposure exercises becoming gradually more challenging each week (see Figure 2). The coach monitors the fear hierarchy and planning and replies with a supportive message once a week for 5 weeks, relevant to the participant’s homework experiences through the secure online platform. All coaching was supervised by an experienced psychotherapist. The intervention is tunneled (ie, no new material is available to the participant until the participant has reported on that week’s achievements and the coach has provided feedback on these achievements). If applicable, the coach sends a standardized reminder message through the secure online platform if the participant did not use the website that week. All actions on the platform (eg, new feedback received, new exercise available) prompted an immediate automated email to the participant. Material from previous weeks remains accessible to the participant. Online coaching messages were delivered through a secured message system on the intervention website by trained and supervised master’s level students of clinical psychology. The participant completes exposure exercises alone and reports on completed exercises weekly. Throughout the intervention period, the participants were kept on the waiting list for face-to-face psychotherapy.

The website platform was migrated to an updated version during the recruitment period. This migration was performed to ensure continuing safety of participant data in accordance with Dutch law and to resolve or mitigate critical bugs and shortcomings in website functionality. Website content, however, remained unaltered throughout recruitment. No substantial website downtime was observed during recruitment.

#### Waiting List Condition

Participants in the waiting list group remained on the waiting list for face-to-face psychotherapy. Additionally, to comply with ethics committee regulations and to provide an incentive for enrolling in the trial, control group participants received a self-help book [40] based on exposure therapy, the de facto standard treatment in phobias. This book was sent to the control group participants free of charge with no instructions or support.

### Assessments

All outcome measures were administered by phone at baseline (T0) and as self-assessment on the Internet at posttest (5 weeks from randomization, T1), using Web-based questionnaire software visibly associated to VU University Amsterdam. This relatively short period was selected to minimize the posttest assessments taking placing during face-to-face psychotherapy if face-to-face psychotherapy should incidentally take place earlier than 6 weeks after inclusion into the trial. To reduce study dropout, intensive reminder emails and telephone reminder calls were used for T1 assessments. Despite several email and telephone reminders, there was considerable variability in follow-up time (mean 50, SD 15.3 days), yet there was no significant difference in follow-up time between the intervention and wait-list control groups. All trial data were stored on a secured network complying with Dutch safety and privacy standards at the time of inclusion and accessible only to research staff. Data were anonymized as soon as possible.

### Outcomes

Outcome measures are described in more detail elsewhere [38].

### Primary Outcome Measures

#### Fear Questionnaire

The primary outcome measure was the Fear Questionnaire (FQ) [42]. This instrument measures severity of fear and avoidance of phobic stimuli. The psychometric validity of the FQ has been established for the Dutch version [43]. Internal consistency was good, with Cronbach alpha ranging from.78 (blood-injection-injury subscale) to 0.84 (total score).

### Secondary Outcome Measures

#### Anxiety

The Beck Anxiety Inventory (BAI) [44] is a 21-item self-report questionnaire that focuses primarily on physiological manifestations of anxiety. The BAI has been validated for patients with agoraphobia [45] and other anxiety disorders [46]. Internal consistency was excellent (Cronbach α=.92).

#### Depressive Symptoms

The Center for Epidemiological Studies-Depression Scale (CES-D) [47] was administered as a self-rated questionnaire on the Internet. A Dutch version of the CES-D has been validated in an Internet-administrated form [48]. Internal consistency in this sample was good (Cronbach α=.70).

### Process Outcome Measures

#### Adherence

Following a recent definition of intended usage [49], we defined *intervention adherence* as “the extent to which individuals should experience the content (of the intervention) to derive maximum benefit.” Because some exercises were deemed to have a larger impact on lowering symptom severity (eg, reporting on performing exposure exercises is more beneficial than filling in a readiness to change questionnaire), different weights were assigned to different exercises accordingly, to a total of 20% for each of the 5 weeks. The intended usage was defined as 100% (ie, finishing the 8 exercises the participants were supposed to finish in 5 weeks). The main use metric was having finished an exercise as verified by the coach.

#### Treatment Satisfaction

Satisfaction with the Internet intervention or the self-help book was evaluated using the Client Satisfaction Questionnaire (CSQ-8) [50] which has been validated for use in a Dutch population [51]. Internal consistency was good (Cronbach α=.85). A few free-text items on participant satisfaction and experiences specific to the Web-based intervention were added.

### Sample Size

To obtain 90% statistical power with a 2-sided alpha equal to .05 and assuming a mean standardized effect size (Cohen’s *d*) of 0.7 in the intervention group and 0.2 in the control group, we calculated that 170 participants were needed to establish a clinical effect of the Internet intervention compared to wait-list controls. Assuming a dropout rate of 30% at 1-year follow-up, 244 participants should be included.

### Randomization

A computer-generated randomization table was prepared by a researcher not involved in the data collection (AvS). Randomization was stratified at clinic level and performed at a 1:1 ratio. To ensure approximately equal randomization ratios per clinic, blocks of 8 were used. An external researcher not involved in the project supervised a list of sequentially numbered allocations and assigned participants to the conditions. All project members involved in data collection were unaware of allocation status until randomization was definitive. Participants were enrolled by a master’s level research assistant.

### Blinding

Due to the nature of this trial, neither participants nor researchers could be blinded to treatment allocation. All outcome measures are self-report questionnaires, which makes blinding unnecessary.

### Statistical Methods

Data were analyzed with SPSS for Windows, version 20 (IBM Corp, Armonk, NY, USA) according to the intention-to-treat (ITT) principle. Using the multiple imputation function implemented in SPSS 20, we imputed missing data at posttest yielding 50 imputed datasets with 50 iterations each using the multiple imputation option with predictive mean matching. Predictors for the imputing procedure were pretest and (nonmissing) posttest scores, as well as age, clinic, education level, gender, randomization status, and quality of life at pretest. Because SPSS does not automatically calculate pooled statistics for imputed datasets when using ANCOVA, we calculated these statistics by pooling the saved residuals from each imputed dataset and reported mean values and 95% confidence intervals. Between-group effects on the primary outcome measure (FQ) at posttest were calculated with an ANCOVA, with baseline scores of the FQ, BAI, and CES-D entered as a covariate. Within- and between-group effect sizes were reported as Cohen’s *d*. Effect sizes of *d*=0.2 are interpreted as small, effect sizes between 0.2 and 0.5 are interpreted as moderate, and effect sizes of 0.8 and upwards are interpreted as large. Due to the large amount of missing data at posttest, data are also presented separately for participants with full follow-up information. No interim analyses were performed. No stopping guidelines were postulated.

### Changes to Protocol

There were no changes from the published study protocol [38].

## Results

### Sample

**Figure 3:**
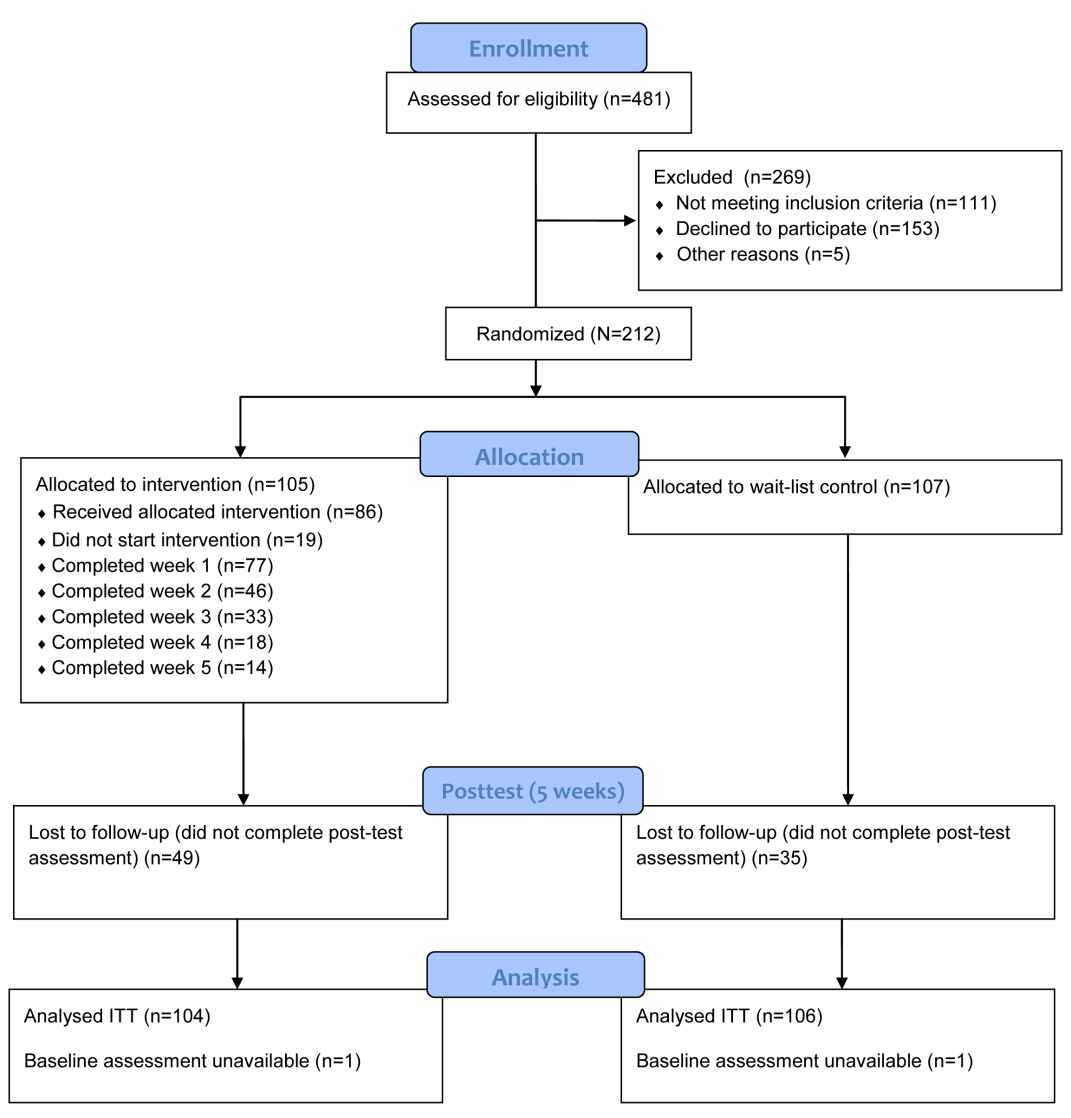
Participant flowchart.

See Figure 3 for a flowchart and overview of participants in this trial. Of 481 participants assessed for eligibility, 212 were randomized to either intervention (n=105) or control (n=107). Baseline data are presented in Table 1. Apart from psychotropic medication use, there were no significant differences between intervention and control groups at baseline. Participants were mainly Dutch, female, and highly educated.

**Table 1:** Baseline characteristics of participants.

Characteristics			Total sample (N=212)	Intervention (n=105)	Control (n=107)	*P* ^a^
**Demographics**						
	Age, mean (SD)		34.6 (11.7)	35.7 (11.7)	33.4 (11.6)	.09
	Female, n (%)		130 (61.0)	58 (55)	72 (67)	.10
	Higher education,^b^ n (%)		120 (57.1)	58 (56)	62 (58)	.69
	Disposable income (€),^c^ mean (SD)		1524 (761)	1545 (727)	1503 (796)	.70
	**Dutch parents, n (%)**					
		Both parents Dutch	144 (68.6)	72 (69)	72 (689)	.98
		One parent Dutch	23 (11)	11 (11)	12 (11)	
		Neither parent Dutch	43 (21)	21 (20)	22 (21)	
	Psychotropic medication, n (%)		43 (20)	14 (13%)	29 (27)	.01
**Baseline scores, mean (SD)**						
	FQ		40.28 (22.71)	42.43 (23.41)	38.19 (21.93)	.24
	BAI		44.81 (13.41)	45.15 (13.76)	44.48 (13.13)	.81
	CES-D		24.82 (8.47)	24.96 (8.61)	24.69 (8.37)	.84
**CIDI phobia diagnoses** ^d^						
	**Specific phobia, n (%)**					
		Animal-type	19 (9)	9 (9)	10 (9)	.84
		Nature-type	35 (17)	21 (20)	14 (13)	.18
		Blood-injection-injury	51 (24)	25 (24)	26 (24)	.93
		Situational-type	72 (34)	38 (36)	34 (32)	.50
		Agoraphobia without panic disorder	36 (17)	18 (17)	18 (17)	.95
		Agoraphobia with panic disorder	87 (41)	48 (45)	40 (37)	.28
		Social phobia	113 (53.3)	51 (49)	62 (58)	.17
	Number of phobias, mean (SD)		1.95 (1.08)	1.99 (1.11)	1.91 (1.06)	.57
	**Number of phobias, n (%)**					
		1 phobia	91 (43)	43 (41)	48 (45)	
		2 phobias	61 (29)	32 (31)	29 (27)	
		≥3 phobias	60 (28)	30 (29)	30 (28)	

^a^ Tested with *t* test or chi-square test as appropriate.

^b^ Equivalent to a Bachelor’s degree or higher.

^c^ N=180.

^d^ Percentages add up to over 100% due to multiple possible diagnoses per participant.

### Study Dropout

Posttreatment assessments were completed by 126 of 212 (59.2%) participants. Due to database corruption, 2 pretreatment assessments were unavailable. We tested whether there were significant differences in all baseline characteristics described in Table 1 between those who completed the follow-up assessments and those who did not, with dropouts scoring significantly higher on the BAI (*t*_210_=2.275, *P*=.02) and the CES-D (*t*_210_=2.489, *P*=.01), and dropouts being younger in age (*t*_210_=2.022, *P*=.04), less often highly educated (χ^2^_210_=8.1, *P*=.004), and taking psychotropic medication less often at the time of assessment (χ^2^_210_=35.4, *P<*.001). Intervention group participants were also more likely to be nonresponders (67%, 84/126 of completers were control group participants; χ^2^_210_=4.3, *P*=.04). Table 2 shows the statistically significant differences between nonresponders and study completers. The differences of other characteristics described in Table 1 were not statistically significant.

**Table 2:** Differences between dropouts and study completers.

Characteristics	Completer (n=126)	Dropout (n=84)	*P* ^a^
Baseline CES-D score, mean (SD)	23.70 (8.34)	26.63 (8.39)	.01
Baseline BAI score, mean (SD)	43.08 (13.54)	47.35 (12.96)	.02
Age, mean (SD)	35.83 (11.82)	32.52 (11.35)	.04
Higher education, n (%)	82 (65%)	38 (45%)	.004
On psychotropic medication, n (%)	43 (34%)	0 (0%)	.<001

^a^ Tested with *t* test or chi-square test as appropriate.

### Intervention Adherence and Satisfaction

Of the 105 participants, 78 (76.4%) started using the intervention, 14 (13.3%) finished week 5, and 9 (8.8%) met the intended usage of 100% (all 8 exercises) in 5 weeks. Average adherence, as expressed as percentage of intended usage, was 37.5% (SD 30.7%); median number of exercises completed (out of a possible 8) was 3 (IQR 4.0). As found in a previous meta-analysis [52], higher education in this sample was associated with higher adherence (*F*_2,103_=8.132, *P*=.005). Intervention participants were moderately positive about their coach (average grade 6.8 of 10, SD 1.06) and indicated that the quality of the feedback messages was satisfactory (10/43, 23%), good (18/43, 42%), or very good to excellent (13/43, 30%). The number of messages received was also evaluated as being balanced (not too many, not too few) by most participants (32/43, 74%). Mean scores for all 8 CSQ-8 items were acceptable (mean 2.78, SD 0.58-0.81; possible item range 1-4).

### Completers and Intention-to-Treat Analyses

**Figure 4:**
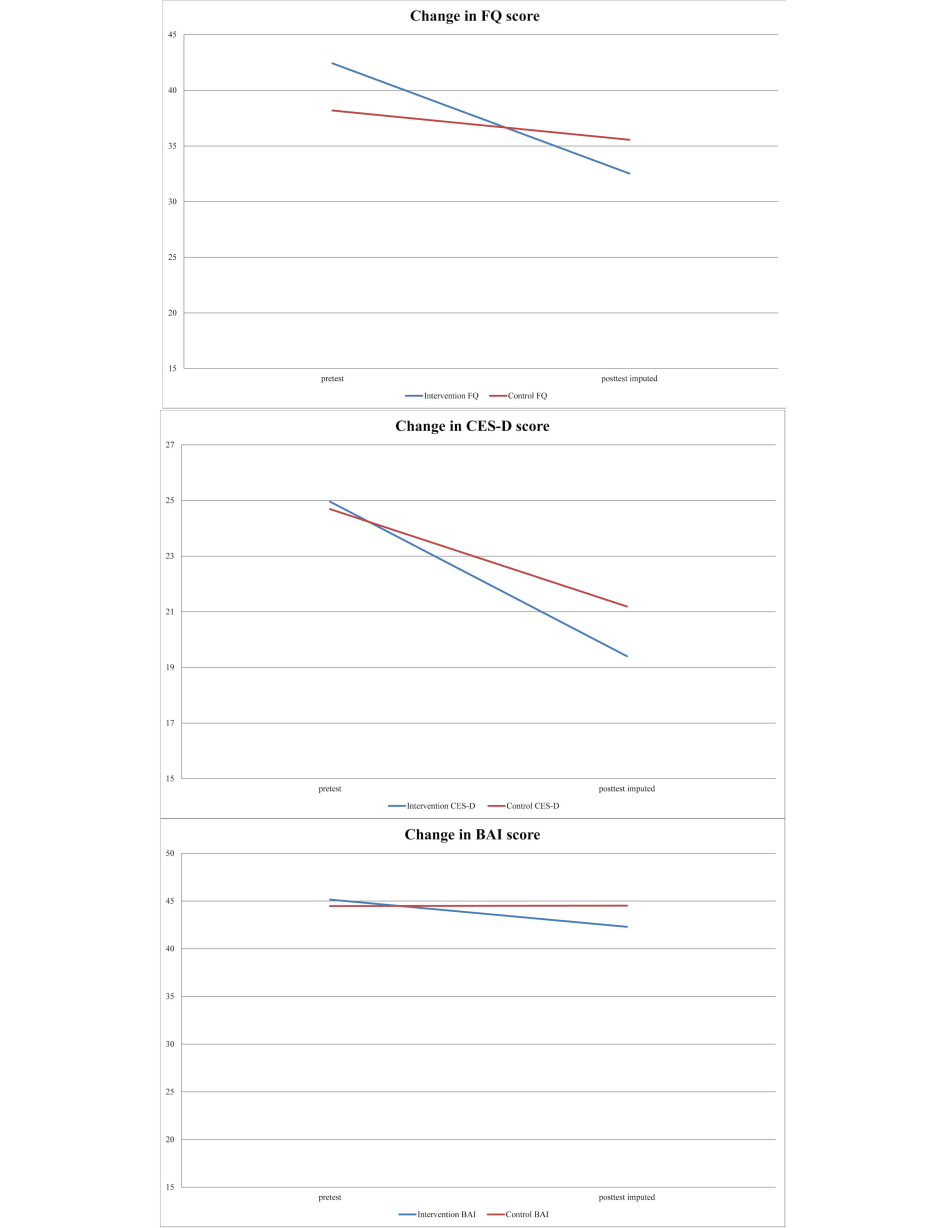
Change in scores between baseline and imputed posttest scores.

After imputing missing values at posttest and correcting for baseline scores of the FQ, BAI, and CES-D, ANCOVA showed a significant difference in FQ scores between intervention and control groups at posttest (*F*_2,208_=6.327, 95% CI 5.977-6.686; *P*=.02, 95% CI .01-.02; partial η^2^=.030, 95% CI .03-.03). ANCOVAs also showed a significant difference in CES-D scores between intervention and control groups at posttest (*F*_2,208_=6.121, 95% CI 5.550-6.669; *P*=.03, 95% CI .02-.03; partial η^2^=.029, 95% CI .03-.03), but no significant difference in BAI scores between intervention and control groups at posttest (*F*_2,208_=4.097, 95% CI 3.818-4.376; *P*=.05, 95% CI .04-.06; partial η^2^=.020, 95% CI .02-.02). Changes in scores are presented in Table 3 and presented graphically in Figure 4.

**Table 3:** Main results, imputed intention-to-treat sample (N=210).

Results		Pretest, mean (SD)	Posttest ITT, mean (SD)	ANCOVA^a^		Effect size, *d* (95% CI)		NNT^b^
				*F*_2,208_	*P*	Within group	Between group	
**Intervention**								
	FQ	42.02 (23.39)	32.52 (18.48)	6.33	.16	0.42 (0.31, 0.52)	0.35 (0.07, 0.62)	5.10
	CES-D	24.99 (8.58)	19.40 (5.97)	6.12	.26	0.75 (0.51, 0.97)	0.34 (0.07, 0.61)	5.26
	BAI	45.01 (13.78)	42.30 (10.68)	4.10	.53	0.22 (0.03, 0.40)	0.28 (0.01, 0.55)	6.41
**Wait-list control**								
	FQ	38.34 (21.97)	35.56 (18.41)	—	—	0.13 (0.03, 0.23)	—	—
	CES-D	24.75 (8.39)	21.19 (7.06)	—	—	0.46 (0.27, 0.65)	—	—
	BAI	44.57 (13.16)	44.52 (10.79)	—	—	0.00 (-0.14, 0.15)	—	—

^a^ Controlled for baseline scores.

^b^ Number needed to treat.

After correcting for baseline scores and age (full follow-ups only), we found no significant differences in posttest scores between intervention and control groups for FQ (*F*_2,208_=.137, *P*=.71, partial η^2^=.001), CES-D (*F*_2,208_=2.086, *P*=.15, partial η^2^=0.017), or BAI (*F*_2,208_=0.333, *P*=.57, partial η^2^=0.003).

### Adverse Events

No adverse events were reported or observed during the trial. In a recent trial, a few participants reported temporary adverse effects in an Internet-based intervention for social anxiety disorder [53]; in particular, exacerbation of anxiety symptoms and negative well-being. One control group participant (1/107, 0.5%) reported a worsening of complaints after having started the intervention. However, it is unknown whether this was due to the intervention or to other circumstances not related to this study.

## Discussion

### Principal Findings

Results show that an Internet-based guided intervention for phobic outpatients can be effective, with modest but significant effect sizes (*d*=0.28-0.35). The between-group effect sizes were small but significant in the imputed sample. Interestingly, effect sizes were similar for the FQ and CES-D. Because spontaneous recovery is common in depression [54] but not in phobia [5,6], it is unlikely that the decrease in phobic complaints can be attributed to spontaneous recovery rather than to the intervention. The intervention was not targeted specifically at depression; therefore, the significant decrease in depressive symptoms as measured by the CES-D might be attributable to spontaneous recovery rather than the intervention, although due to randomization this spontaneous recovery should probably have occurred in the control condition as well. Another possible explanation is the decrease in phobic complaints in the intervention group led to a commensurate decrease in depressive symptoms. Because the control group participants received a self-help book as compensation for their time invested in completing the baseline and follow-up assessments, there is a possibility that the between-group effect sizes are a more conservative estimate of the real effect size. Additionally, effect sizes for the BAI were lowest of all 3 primary outcome measures. This may be a result of the BAI being concerned mostly with those physical sensations often associated with panic attacks rather than phobias.

### Comparison to Earlier Literature

Compared with earlier research, recruitment of patients in the outpatient clinics seemed to be reasonably successful. Of the 481 patients assessed for eligibility, of 370 eligible patients we randomized 212 of the 244 participants intended. In all, 153 patients (31.8%) declined to participate in the study (before being screened) and 111 (23.1%) did not meet inclusion criteria. In total, we were able to include 44.1% of all referrals with approximately 2 patients needed to be screened to include a single participant and approximately 3 hours required for each included participant. This does, however, not include the amount of time needed at the outpatient clinic for administrative tasks. In contrast, a recent study of computerized CBT for anxiety in secondary care [55] managed to randomize only 8% (88 of 1141) of referrals. The recruitment percentage also compares rather favorably to trial recruitment in primary care, which can be problematic in terms of duration and numbers of participants recruited as compared to the planned recruitment time and numbers of participants [56]. For example, 1 study focusing on Internet-delivered CBT for depression managed to recruit only 7 patients from 11 general practices in 8 months [57], a marked contrast to our trial. This may be a result of keeping close contact with outpatient staff, although it is difficult to draw comparisons because similar studies with tight integration in routine outpatient clinics are scarce and health care systems differ on national levels. When comparing effect sizes of the current trial with earlier trials of Internet-based anxiety and phobia interventions, we found that the effect sizes of the current trial are low overall [19,58]. In face-to-face psychotherapy, effect sizes of psychological treatment for participants meeting diagnostic criteria were found to be lower than those not meeting the criteria (eg, [13]), but another study found large and sustained effects of Internet-based CBT for panic disorder with and without agoraphobia in routine psychiatric care [33]. Although these previous trials used CBT compared to exposure therapy, it should be noted that the intervention of the current trial included a number of CBT components and that there is no unambiguous evidence that CBT should outperform exposure therapy for phobias per se, with studies finding either no difference or a negligible advantage for either CBT of exposure therapy in phobias (eg, [14,19,59,60]). There is an exception for social anxiety disorder, where cognitive therapies appear to outperform exposure therapy [13,61,62]. It should be noted that just over half of the participants in the current trial were diagnosed with social anxiety disorder, indicating that perhaps greater attention should have been paid to the cognitive therapy elements in the intervention. However, comorbidity of different phobias was large in the current trial, and participants suffering from multiple phobias were encouraged to focus on a single, well-circumscribed area of phobic avoidance at the start of the intervention. Many participants explicitly chose to work on specific phobias or agoraphobia rather than social anxiety, indicating that exposure therapy was indeed the right choice for these participants.

Overall, few (n=9) participants completed all 8 exercises, and 14 completed all 5 weeks, indicating that some exercises were skipped by the participants. Ending a treatment early may not necessarily be a negative finding [63]. Although not many participants stated a reason for not finishing the intervention, the primary reasons given were a lack of time (8 of 27) and the intervention not being suited to the needs of the participant (5 of 27), both commonly cited barriers to the uptake of online interventions [64]. Despite the high acceptability, as expressed by the low percentage of participants refusing to enter the trial outright, low adherence to this intervention remains a cause for concern because it seems to be lower than generally found in other Internet interventions for anxiety disorders [64]. Possible causes for the lower adherence in this intervention may be that the intervention was too broad, targeting all types of phobias within 1 intervention. Additionally, participants were aware that they would receive face-to-face psychotherapy regardless of whether they finished the online intervention, which may have lowered their motivation to persist. Furthermore, the intervention itself may not have been persuasive enough to encourage repeated use [49]. Earlier efforts to identify predictors of treatment dropout in social anxiety disorder, for example, have not yielded consistent results [65], and future research into the causes of premature treatment termination is needed.

A particular strength of this study is the high acceptance rate among outpatients as compared with other studies in outpatients and specialized health care centers, which indicates that this sample is clinically relevant and that the results may generalize well across other outpatient samples using similar recruitment strategies. Implementation in routine practice would perhaps facilitate better uptake due to dropping the constraints surrounding research-oriented RCT setting (eg, randomization, filling in extra questionnaires).

### Limitations

A number of limitations should be taken into account when interpreting the results from this trial. Firstly, the number of participants fell slightly short from the target number of participants (212 randomized versus 244 targeted). Far-reaching cutbacks in Dutch mental health care during the recruitment into this trial resulted in a dwindling number of patients seeking help with outpatient clinics, effectively shrinking the overall participant pool. Secondly, recruitment through outpatient clinics depends on outpatient clinic staff and may be liable to selection bias. Although the included sample of participants seems relatively representative of a clinical sample, selection bias may have occurred during the outpatient clinics’ own selection procedures over which we had no influence. Thirdly, although we corrected for missing values at follow-up by using multiple imputation, the results should be interpreted with caution due to the large amount of missing data. Imputation is an approximation based on a combination of chosen predictors, techniques, and imputing algorithms, which may yield varying results in different datasets [66], making extensive sensitivity analyses time consuming and inconclusive at best. Although some argue that using covariates yields similar results to imputing [67], this may depend heavily on the dataset, and multiple imputation remains the solution of choice for missing data [68]. Regardless of the method for accounting for missing data, the large amount of missing data in this trial is a limitation and means that results should be interpreted with caution. Thirdly, there was considerable variability in the time between baseline assessment and follow-up (median 48 days, range 29-138 days). However, there were no significant correlations between baseline and posttest scores and the time in days between baseline assessment and posttest assessment, which indicates that the posttest scores as used are a representative assessment. Finally, offering the control group participants a self-help book may have influenced the between-group results because wait-list participants using the self-help book may have improved. However, because an improvement in control group participants would mean a smaller contrast between groups, this would lead to a more conservative estimate of treatment effects. Furthermore, it has been proposed that the use of a wait-list group design may not be a representative control group in that it is functionally different from a no-treatment group. It has also been put forward as actually being a “nocebo” group in that waitlisted participants actually do worse than no-treatment participants [69], which would theoretically lead to an inflation of between-group effect sizes. In the light of the current trial, however, both intervention and control groups were scheduled to receive face-to-face psychotherapy; as such, the arguments pertaining to possibly higher (or lower) effect sizes when using a wait-list control group do not necessarily apply to the current trial.

### Implications and Future Research

In summary, adding an online guided intervention to routine face-to-face treatment may prove beneficial for outpatients, regardless of type of phobia diagnosis. However, effect sizes were markedly lower (*d*=0.28-0.35) than those found in research on psychological treatment for phobias in the general population (*d*=0.70-1.84) [12-14] and for anxiety in primary care (*d*=0.57) [70], and were only found for the imputed ITT sample. Because there was systematic attrition in this trial, the significant differences between completers and dropouts may provide valuable information to identify focal points for targeted attrition reduction, although previous efforts to identify nontechnological factors influencing attrition in online interventions have yielded inconclusive results [71-75]. Given the combination of high acceptability and low adherence, future research should focus on optimizing the usability and persuasive design of this intervention to improve retention and adherence [49] to maximize potential benefits of an intervention that efficiently uses the time spent waiting for face-to-face psychotherapy. Independent replication of the current results in different outpatient settings and countries is needed to verify the findings before robust inferences can be made, but the current results are promising.

## Multimedia Appendix 1

CONSORT-EHEALTH checklist V1.6.2 [76].

## Abbreviations

BAI: Beck Anxiety Inventory
CBT: cognitive behavioral therapy
CIDI: Composite International Diagnostic Interview
FQ: Fear Questionnaire
GP: general practitioner
IQR: Interquartile range
ITT: intention-to-treat
NNT: number needed to treat
RCT: randomized controlled trial
