# Supplementary material for: Short-Term Effectiveness of Web-Based Guided Self-Help for Phobic Outpatients: Randomized Controlled Trial
Source: J Med Internet Res. 2014 Sep 29;16(9):e226. doi: 10.2196/jmir.3429 (PMC4211034; doi:10.2196/jmir.3429)
Supplement: Supplementary file 1 [file jmir_v16i9e226_app1.pdf]

CONSORT-EHEALTH Checklist V1.6.2 Report

(based on CONSORT-EHEALTH V1.6), available at [http://tinyurl.com/consort-ehealth-v1-6].

3429

Date completed

3/26/2014 8:04:46

by

Robin Kok

Short-term effectiveness of web-based guided self-help for phobic outpatients: results from a randomised controlled trial

TITLE

1a-i) Identify the mode of delivery in the title

"Short-term effectiveness of web-based guided self-help for phobic outpatients: results from a randomised controlled trial"

Web-based is mentioned unambiguously in the title.

1a-ii) Non-web-based components or important co-interventions in title

Yes - no offline co-interventions so not needed in title.

1a-iii) Primary condition or target group in the title

"Short-term effectiveness of web-based guided self-help for phobic outpatients: results from a randomised controlled trial"

Yes - mentions phobic outpatients.

ABSTRACT

1b-i) Key features/functionalities/components of the intervention and comparator in the METHODS section of the ABSTRACT

Yes, mentioned in the abstract are "a five-week internet-based guided self-help programme based on exposure therapy followed by FtF psychotherapy" and comparator "wait-list control group (WLC) followed by FtF psychotherapy".

1b-ii) Level of human involvement in the METHODS section of the ABSTRACT

Yes - abstract mentions "weekly student support".

1b-iii) Open vs. closed, web-based (self-assessment) vs. face-to-face assessments in the METHODS section of the ABSTRACT

Yes - "Assessments took place by telephone at baseline (T0) and on the Internet at post-test (T1, self-assessment at five weeks after baseline)."

1b-iv) RESULTS section in abstract must contain use data

Yes - (verbatim from Abstract): "At post-test, analysis of covariance on the intention-to-treat sample showed significant but small effect sizes between intervention and control groups on the FQ (d = 0.35, p = .016), CES-D (d = 0.34, p = .026), and a near-significant effect size on the BAI (d = 0.28. p = .053). However, high non-response was observed, with 86 participants (40.8%) lost to follow-up at T1 and only 14 (13.3%) participants finishing all five weeks."

1b-v) CONCLUSIONS/DISCUSSION in abstract for negative trials

N/A - not a negative trial

INTRODUCTION

2a-i) Problem and the type of system/solution

Verbatim: "Phobias are among the most common mental disorders and the most common type of anxiety disorders (...) Internet-based interventions are increasingly popular adaptations of evidence based psychotherapies as a replacement of, or adjunct to traditional face to face (FTF) therapies. (...) Only limited research on internet-based psychological treatment of anxiety disorders or phobias in outpatients has been done. There appear to be no large scale, high quality trials evaluating the efficacy of internet-based exposure therapy in phobic outpatients (...) The objective of the current trial was to assess the short-term clinical effectiveness of offering internet-based guided self-help to outpatients compared to a wait-list control.

2a-ii) Scientific background, rationale: What is known about the (type of) system

Yes - "In past years, Internet interventions have been found efficacious for all phobias, like agoraphobia, e.g. anxiety disorders, and more specifically agoraphobia, specific phobias and social phobia. Thus, internet-delivered psychological treatments for anxiety and phobias show a promise of being feasible, acceptable and effective."

METHODS

3a) CONSORT: Description of trial design (such as parallel, factorial) including allocation ratio

"As wait-lists are commonplace in outpatient clinics, time spent waiting for FtF treatment could be spent effectively by offering a (guided) self-help intervention to patients. Delegating the routine, basic elements of exposure treatment to a guided Internet-based situation could shorten FtF therapy , making the treatment more cost-effective. "

3b) CONSORT: Important changes to methods after trial commencement (such as eligibility criteria), with reasons

Yes - "There were no changes from the published study protocol"

3b-i) Bug fixes, Downtimes, Content Changes

Yes - "The website platform was migrated to an updated version during the recruitment period. This migration was performed to ensure continuing safety of participant data in accordance with Dutch law and to resolve or mitigate critical bugs and shortcomings in website functionality. Website content, however, remained unaltered throughout recruitment. No substantial website downtime was observed during recruitment."

4a) CONSORT: Eligibility criteria for participants

Yes - All computer-literate patients with a possible phobia (social phobia, agoraphobia or specific phobia) were referred to the researchers by the outpatient clinic, even if a phobia was not the primary reason for seeking treatment at an outpatient clinic. Participants had to be (a) 18 or over, (b) currently enrolled to receive FtF psychotherapy at one of the participating outpatient clinics, (c) having a DSM-IV-TR or ICD-10 diagnosis of any phobia as established by the CIDI. Psychotropic medication use was allowed if stable for at least the duration of the intervention or control group period. Patients presenting with psychotic disorders or at elevated risk for suicide were excluded from the trial but remained on the wait-list for FtF psychotherapy at their outpatient clinics.

4a-i) Computer / Internet literacy

Yes - computer literacy explicitly mentioned

4a-ii) Open vs. closed, web-based vs. face-to-face assessments:

Yes - "All computer-literate patients "(...) referred to the researchers by the outpatient clinic"

4a-iii) Information giving during recruitment

No - available in referenced protocol article

4b) CONSORT: Settings and locations where the data were collected

Yes - "Eight specialised anxiety disorder outpatient clinics in medium to large cities in the west of the Netherlands participated"

4b-i) Report if outcomes were (self-)assessed through online questionnaires

Yes - "All outcome measures were administered by phone at baseline (T0) and as self-assessment on the internet at post-test (5 weeks from randomisation, T1)."
4b-ii) Report how institutional affiliations are displayed
Yes - "using web-based questionnaire software visibly associated to VU University Amsterdam."
5) CONSORT: Describe the interventions for each group with sufficient details to allow replication, including how and when they were actually administered
5-i) Mention names, credential, affiliations of the developers, sponsors, and owners
Yes - "The Internet-based intervention is an adaptation of an existing self-help book on phobias (ref)"
5-ii) Describe the history/development process
N/A
5-iii) Revisions and updating
No dynamic content / no version numbers used
5-iv) Quality assurance methods
Intervention content was adapted from an existing book / intervention coaches were supervised by licensed psychotherapist: "All coaching was supervised by an experienced psychotherapist"
5-v) Ensure replicability by publishing the source code, and/or providing screenshots/screen-capture video, and/or providing flowcharts of the algorithms used
Yes - Screenshots included as appendices.
5-vi) Digital preservation
Yes
5-vii) Access
N/A
5-viii) Mode of delivery, features/functionalities/components of the intervention and comparator, and the theoretical framework
"The intervention is offered at no cost to the participant, takes five weeks to complete and is based on psycho-education and exposure therapy. The broad and non-specific focus of the intervention is on identifying and correcting avoidance behaviour by using exposure, a common and evidence-based therapeutic component of most phobia therapies. This broad focus facilitates using the intervention for the entire range of phobias."
5-ix) Describe use parameters
Yes - "Following a recent definition of 'intended usage', we defined intervention adherence as 'the extent to which individuals should experience the content (of the intervention) to derive maximum benefit'. As some exercises were deemed to have a larger impact on lowering symptom severity (e.g., reporting on performing exposure exercises is more beneficial than filling in a readiness to change questionnaire), different weights were assigned to different exercises accordingly to a total of 20% for each of the five weeks. The intended usage was defined as 100%, i.e. finishing all 8 exercises in all 5 weeks. "
5-x) Clarify the level of human involvement
Yes - "The coach monitors the fear hierarchy and planning and replies with a 5 weekly supportive messages relevant to the participant's homework experiences through the secure online platform"
5-xi) Report any prompts/reminders used
Yes - "If applicable, the coach sends a standardized reminder message if the participant did not use the website that week (...) All actions on the platform (e.g., new feedback received, new exercise available) prompted an immediate automated e-mail to the participant."
5-xii) Describe any co-interventions (incl. training/support)
N/A
6a) CONSORT: Completely defined pre-specified primary and secondary outcome measures, including how and when they were assessed
Yes - primary outcomes fully described
6a-i) Online questionnaires: describe if they were validated for online use and apply CHERRIES items to describe how the questionnaires were designed/deployed
Yes - all questionnaires validated for online use
6a-ii) Describe whether and how "use" (including intensity of use/dosage) was defined/measured/monitored
Yes - "The main usage metric was having finished an exercise, as verified by the coach"
6a-iii) Describe whether, how, and when qualitative feedback from participants was obtained
Yes - "A few items free-text items on participant satisfaction and experiences specific to the web-based intervention were added."
6b) CONSORT: Any changes to trial outcomes after the trial commenced, with reasons
Yes - None
7a) CONSORT: How sample size was determined
7a-i) Describe whether and how expected attrition was taken into account when calculating the sample size
Yes - "To obtain 90% statistical power with a 2-sided α equal to 0.05 and assuming a mean standardised effect size (Cohen's d) of 0.7 in the intervention group and 0.2 in the control group, we calculated that 170 participants were needed to establish a clinical effect of the internet intervention compared to WLC. Assuming a dropout rate of 30% at one-year follow-up, thus, 244 participants should be included."
7b) CONSORT: When applicable, explanation of any interim analyses and stopping guidelines
Yes - no interim analysis or stopping guidelines were employed: "No interim analyses were performed. No stopping guidelines were postulated."
8a) CONSORT: Method used to generate the random allocation sequence
Yes - "A computer-generated randomisation table was prepared by a researcher not involved in the data collection"
8b) CONSORT: Type of randomisation; details of any restriction (such as blocking and block size)
Yes - "To ensure approximately equal randomisation ratios per clinic, blocks of eight were used."
9) CONSORT: Mechanism used to implement the random allocation sequence (such as sequentially numbered containers), describing any steps taken to conceal the sequence until interventions were assigned
Yes - "An external researcher not involved in the project supervised a list of sequentially numbered allocations and assigned participants to the conditions."
10) CONSORT: Who generated the random allocation sequence, who enrolled participants, and who assigned participants to interventions
Yes - "A computer-generated randomisation table was prepared by a researcher not involved in the data collection"
11a) CONSORT: Blinding - If done, who was blinded after assignment to interventions (for example, participants, care providers, those assessing outcomes) and how
11a-i) Specify who was blinded, and who wasn't
Yes - "Due to the nature of this trial, neither participants nor researchers could be blinded to treatment allocation. All outcome measures are self-report questionnaires which makes blinding unnecessary."

11a-ii) Discuss e.g., whether participants knew which intervention was the "intervention of interest" and which one was the "comparator"
Yes - "...participants (...) could [not] be blinded to treatment allocation"
11b) CONSORT: If relevant, description of the similarity of interventions
N/A
12a) CONSORT: Statistical methods used to compare groups for primary and secondary outcomes
Yes - "Between-groups effects on primary outcome measure (FQ) at post-test were calculated with an ANCOVA, with baseline scores of the FQ, BAI and CES-D entered as a covariate."
12a-i) Imputation techniques to deal with attrition / missing values
Yes - "Using the multiple imputation function implemented in SPSS 20, we imputed missing data at post-test, yielding 50 imputed datasets with 50 iterations each using the multiple imputation option with predictive mean matching in SPSS 20. Predictors for the imputing procedure were pre-test and (non-missing) post-test scores, as well as age, clinic, education level, gender, randomisation status and quality of life at pre-test. Since SPSS does not automatically calculate pooled statistics for imputed datasets when using ANCOVA, we calculated these statistics by pooling the saved residuals from each imputed data set and report mean values and 95% confidence intervals."
12b) CONSORT: Methods for additional analyses, such as subgroup analyses and adjusted analyses
N/A

RESULTS

13a) CONSORT: For each group, the numbers of participants who were randomly assigned, received intended treatment, and were analysed for the primary outcome
Yes - flowchart included
13b) CONSORT: For each group, losses and exclusions after randomisation, together with reasons
Yes - flowchart according to CONSORT guidelines is included
13b-i) Attrition diagram
Yes - incorporated in CONSORT flowchart
14a) CONSORT: Dates defining the periods of recruitment and follow-up
Yes - "Recruitment period commenced in August 2010 and was concluded in December 2013"
14a-i) Indicate if critical "secular events" fell into the study period
Not reported
14b) CONSORT: Why the trial ended or was stopped (early)
Yes - "[recruitment] (...) was stopped in December 2013 to allow for sufficient follow-up time."
15) CONSORT: A table showing baseline demographic and clinical characteristics for each group
Yes - in table
15-i) Report demographics associated with digital divide issues
Yes - in table
16a) CONSORT: For each group, number of participants (denominator) included in each analysis and whether the analysis was by original assigned groups
16-i) Report multiple "denominators" and provide definitions
Yes - in manuscript (results)
16-ii) Primary analysis should be intent-to-treat
Yes - ITT used
17a) CONSORT: For each primary and secondary outcome, results for each group, and the estimated effect size and its precision (such as 95% confidence interval)
Yes - see results
17a-i) Presentation of process outcomes such as metrics of use and intensity of use
Yes - see results
17b) CONSORT: For binary outcomes, presentation of both absolute and relative effect sizes is recommended
N/A
18) CONSORT: Results of any other analyses performed, including subgroup analyses and adjusted analyses, distinguishing pre-specified from exploratory
N/A
18-i) Subgroup analysis of comparing only users
N/A
19) CONSORT: All important harms or unintended effects in each group
Yes - "No adverse events were reported or observed during the trial. In a recent trial, a few participants reported passing side effects in an Internet-based intervention for social anxiety disorder; in particular exacerbation of anxiety symptoms and negative well-being. One control group participant (0.5%) reported a worsening of complaints."
19-i) Include privacy breaches, technical problems
N/A
19-ii) Include qualitative feedback from participants or observations from staff/researchers
N/A

DISCUSSION

20) CONSORT: Trial limitations, addressing sources of potential bias, imprecision, multiplicity of analyses
20-i) Typical limitations in ehealth trials
Yes - see limitations
21) CONSORT: Generalisability (external validity, applicability) of the trial findings
21-i) Generalizability to other populations
Yes - "A particular strength of this study is the high acceptance rate among outpatients as compared to other studies in outpatients and specialised health care centres, which indicates that this sample is clinically relevant and that the results may generalise well across other outpatient samples using similar recruitment strategies."
21-ii) Discuss if there were elements in the RCT that would be different in a routine application setting
Yes - "Implementation in routine practice would perhaps facilitate better uptake due to dropping the constraints surrounding research-oriented RCT setting (e.g., randomisation, filling in extra questionnaires, etc.) "
22) CONSORT: Interpretation consistent with results, balancing benefits and harms, and considering other relevant evidence

22-i) Restate study questions and summarize the answers suggested by the data, starting with primary outcomes and process outcomes (use)
Yes - see discussion
22-ii) Highlight unanswered new questions, suggest future research
Yes - see discussion

Other information

23) CONSORT: Registration number and name of trial registry
Yes - "Netherlands Trial Register NTR2233 "
24) CONSORT: Where the full trial protocol can be accessed, if available
Yes - Published elsewhere, referenced several times throughout manuscript, freely available online without subscription.
25) CONSORT: Sources of funding and other support (such as supply of drugs), role of funders
Yes - "This study is funded by ZonMw (project number 80-82310-97-10015). The funding body had no influence in the design, execution, analysis or interpretation of the results of this study."
X26-i) Comment on ethics committee approval
Yes - "This trial was approved by the Medical Ethics Committee of the VU Medical Centre, Amsterdam (registration number 2010/77)"
x26-ii) Outline informed consent procedures
Yes - "after (...) obtaining informed consent in writing"
X26-iii) Safety and security procedures
Yes - "All trial data were stored on a secured network complying with Dutch safety and privacy standards at the time of inclusion."
X27-i) State the relation of the study team towards the system being evaluated
No competing interests declared.
